# Conditioned taste aversion in the cricket *Gryllus bimaculatus*

**DOI:** 10.1038/s41598-022-13500-x

**Published:** 2022-06-13

**Authors:** Hui Lyu, Makoto Mizunami

**Affiliations:** 1 Graduate School of Life Science, Hokkaido University, Sapporo, 060-0810 Japan; 2 Faculty of Science, Hokkaido University, Sapporo, 060-0810 Japan

**Keywords:** Neuroscience, Psychology

## Abstract

Conditioned taste aversion (CTA) is a form of classical conditioning in which animals associate the taste of a food with illness caused by toxin contained in the food. CTA in mammals is achieved with a long interval of up to several hours between food ingestion and illness induced by LiCl injection. Insects also exhibit CTA, but not much is known about its features. We investigated whether the cricket *Gryllus bimaculatus* exhibits CTA when ingestion of a sugar solution is followed by LiCl injection. Crickets that ingested sucrose solution 5–10 min before LiCl injection exhibited reduction of sucrose consumption tested 24 or 48 h after injection compared to that tested 24 h before injection. In contrast, crickets that ingested sucrose solution 5–10 min after LiCl injection or 1 h or 8 h before or after injection did not exhibit reduction of sucrose consumption, indicating that reduction of sucrose consumption by CTA training is pairing-specific. We conclude that CTA in crickets is similar to that in mammals in that one-trial pairing is sufficient to achieve memory retention for days, but it differs in that it is achieved with a relatively short interval (< 1 h) between food ingestion and toxin injection.

## Introduction

Learning to avoid ingestion of toxin-containing foods is essential for survival of animals, especially for omnivores, and hence many animals including humans^1^ exhibit aversion to the taste of food when it contained toxin that produced a negative visceral reaction or when ingestion of food was followed by injection of toxin into the circulation system. This learning is called conditioned taste aversion (CTA) and is characterized as a form of Pavlovian conditioning in which the taste serves as conditioned stimulus (CS) and malaise or illness produced by toxin serves as unconditioned stimulus (US)^2,3^. CTA was first established in rats, and extensive studies in rats demonstrated that CTA has special features that are rarely observed in standard Pavlovian conditioning systems. The first is that one trial conditioning is sufficient for the formation of CTA and the memory lasts 18 days or more^4,5^. The second is that conditioning is achieved with a very long interval of up to several hours between taste stimulation and lithium chloride (LiCl) injection^6^. The third is that CTA is achieved more easily with a novel taste than with tastes of daily foods^7^. The fourth is that the illness is easily associated with taste of food but not with the color or shape of the food^8^. Illness is also associated with the odor of the food when it is compounded with a taste of the food^7,9^. Attempts have been made to clarify physiological and neural mechanisms of CTA in rats, but much remains to be elucidated^10^.

CTA has also been reported in invertebrates including molluscs^11–13^ and insects (moth larvae^14^; locusts^15,16^; honey bees^17,18^; fruit-flies^19^) and there are some reports on mechanisms conveying post-ingestive aversive signals (honey bees^17^; fruit-flies^19^). In insects, however, little effort has been directed to the elucidation of conditioning parameters for achieving CTA, and it therefore remains unclear whether CTA in insects has features analogous to those in mammals. For example, it remains unknown whether CTA can be achieved with an interval of 1 h or longer between food intake and toxin injection into the haemolymph in insects, which is a prominent feature of CTA in mammals.

Aversive learning of the odor of food after ingestion of harmful substances has been reported in honey bees^20,21^, fruit-flies^19^ and locusts^22^. In a study in locusts (*Schistocerca americana*), stimulus parameters for achieving odor aversion learning were investigated by pairing ingestion of odorous food with injection of a toxin^22^. However, it is unknown whether features of this odor learning match those of taste aversion learning in insects.

In this study, we first investigated whether CTA is achieved in the cricket *Gryllus bimaculatus,* a generalist omnivore as in the case of rats, by pairing ingestion of sugar solution with injection of LiCl into the haemolymph, and we then investigated stimulus parameters necessary for achieving CTA. Crickets have emerged as useful experimental insects for investigating the basic associative processes that underlie Pavlovian conditioning^23–27^ and may serve as experimental animals for investigating physiological and neural processes underlying CTA.

## Results

### Determination of the appropriate LiCl concentration for CTA experiments

**Figure 1:**
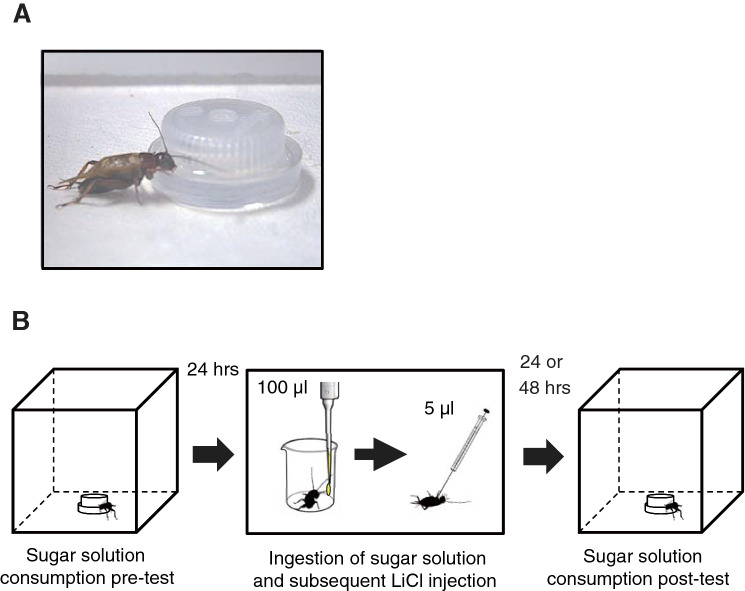
Experimental procedures for CTA training and testing in crickets. (**A**) A feeder used for the sugar consumption test. (**B**) CTA training to ingest 100 µl sugar solution followed by injection of 5 µl of 1 M LiCl solution. The amount of consumption of sugar solution for a given period of time was measured 24 h before CTA training and 24 or 48 h after CTA training.

**Figure 2:**
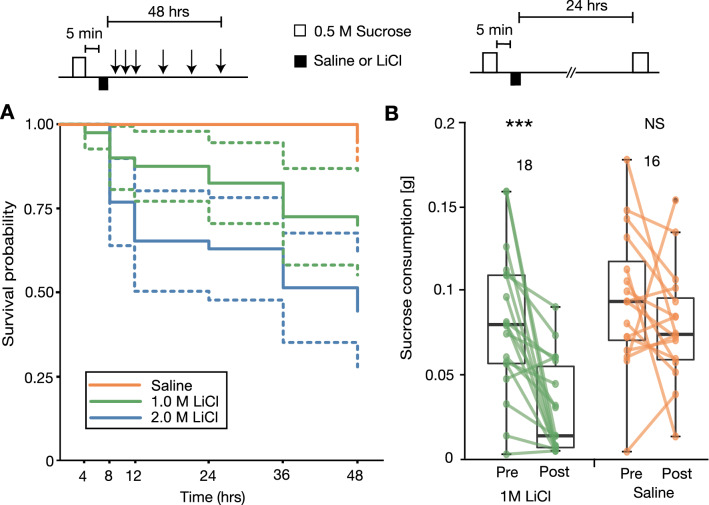
Effects of injections of different concentrations of LiCl solution paired with sucrose consumption. (**A**) Kaplan–Meier plots of survival probabilities of three groups of crickets that received injection of different concentrations of LiCl solution. Crickets in all groups were allowed to consume 0.5 M sucrose solution for 2.5 min and they were injected 5 min later with 5 µl of saline (n = 50) or saline containing 1 M LiCl (n = 50) or 2 M LiCl (n = 54) solution. The probability of survival was measured at various times up to 48 h after injection. Dashed lines indicate 95% confidence interval. The log-rank test was used to compare survival probabilities of different groups. (**B**) Two groups of crickets were allowed to consume 0.5 M sucrose solution for 2.5 min (pre-test) and they were injected 5 min later with 5 µl of saline or saline containing 1 M LiCl. Sucrose consumption was tested again for 2.5 min 1 day after injection (post-test). The Wilcoxon test was used for statistical comparisons of amounts of sucrose consumption before and after the CTA training (*** P < 0.001, ns: not significant).

We first performed experiments to determine the appropriate concentration of LiCl for CTA experiments. Three groups of crickets were allowed to consume 0.5 M sucrose solution from a feeder for 2.5 min (Fig. 1A). Five minutes later, these groups were each injected with 5 µl of cricket saline (n = 50) or saline containing 1 M LiCl (n = 50) or 2 M LiCl (n = 54) into the haemolymph. At various times from 4 to 48 h after injection, the probability of survival of crickets was recorded. Survival analysis using Kaplan–Meier curves (Fig. 2A) showed that the survival probability significantly differed among the three groups (log-rank test: χ2 = 23.68, df = 2, p < 0.0001). Comparison between two groups showed that the survival probability of the saline group was significantly higher than that of the 1 M LiCl group (log-rank test with Bonferroni correction, p = 0.0099) and that of the 2 M LiCl group (p < 0.0003), indicating that survival probability is reduced in the LiCl groups. The survival probability of the 2 M LiCl group was lower at any time after injection than that of the 1 M LiCl group, but the difference was not statistically significant (log-rank test with Bonferroni correction, p = 0.0852). Considering the results, we injected 5 µl of 1 M LiCl solution in subsequent CTA experiments.

We next studied the effect of pairing of ingestion of sucrose solution with LiCl injection on consumption of sucrose solution tested 24 h after the pairing. Two groups of crickets were allowed to consume 0.5 M sucrose solution from a feeder for 2.5 min. Five minutes later, they were injected with 5 µl of saline or saline containing 1 M LiCl solution. At 24 h after injection, they were tested with the amount of consumption of 0.5 M sucrose solution by presenting a feeder for 2.5 min. Crickets that did not visit the feeder and hence experienced no sugar taste in the test were not used for data analysis in this and in subsequent experiments. This was because we intended to evaluate the amount of sugar consumption of crickets that perceived sugar by touching the solution with their mouth or palpi. Crickets that did not visit the feeder accounted for 6% (2/36) of the crickets used in this experiment. The amount of consumption in the final post-training test did not significantly differ from that in the initial pre-training test in the saline-injected group (n = 16, Wilcoxon test, p = 0.27, Fig. 2B). On the other hand, the LiCl-injected group exhibited a significant reduction of sucrose consumption in the final test compared to that in the initial test (n = 18, Wilcoxon test, p = 0.00074, Fig. 2B). Whether the reduction of sucrose consumption is due to association of sucrose taste with the toxic effect of LiCl or due to a non-associative toxic effect of LiCl injection to reduce motivation for intake fluid was the subject of subsequent experiments.

### Effects of pairing sucrose consumption and LiCl injection with different intervals

We next investigated whether crickets exhibit aversion to sucrose when there is long interval between sucrose ingestion and LiCl injection or when crickets received LiCl injection and then ingested sucrose solution. In this experiment, crickets were allowed to ingest a given amount (100 µl) of 0.5 M sucrose solution in CTA training to reduce variance of sensory experience in training among individuals (Fig. 1B). Six groups of crickets received an initial consumption test of sucrose solution for 2.5 min, and the next day they were given 100 µl of sucrose solution 8 h, 1 h or 5–10 min before LiCl injection (8 h, 1 h and 5–10 min before groups) or 5–10 min, 1 h or 8 h after LiCl injection (5–10 min, 1 h and 8 h after groups). The interval between the initial test and LiCl injection was 24 h in this experiment and in all subsequent experiments. At 24 h after LiCl injection, all groups were given a final consumption test of sucrose solution.

**Figure 3:**
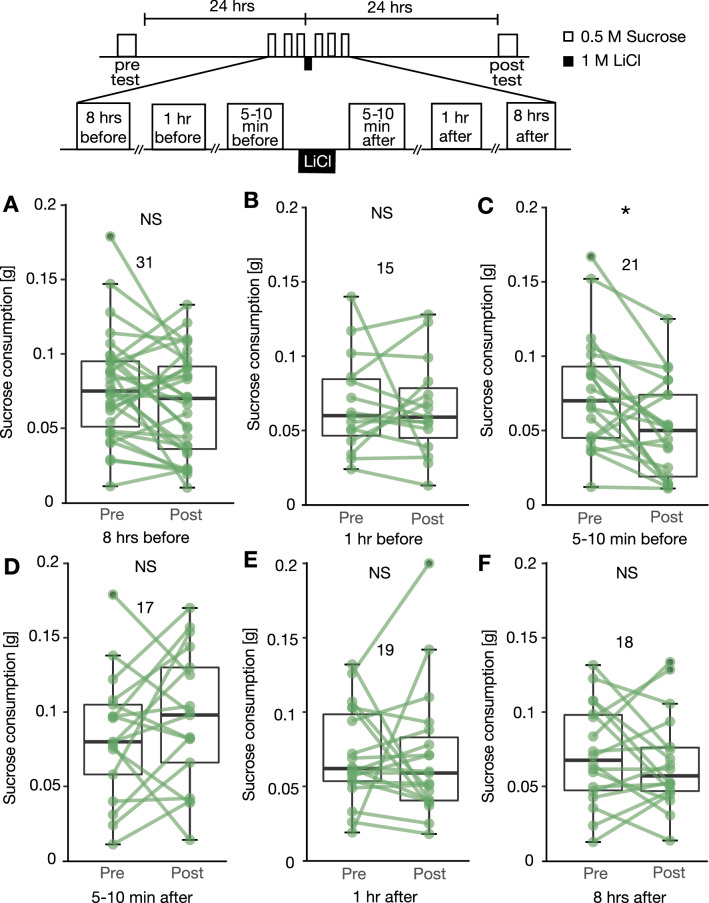
Effects of CTA training with different intervals between sucrose ingestion and LiCl injection. Five groups of crickets received an initial 2.5-min consumption test, and the next day they were allowed to ingest 100 µl of 0.5 M sucrose solution at various times before and after injection of LiCl. All of the groups received 2.5-min sucrose consumption at 24 h after LiCl injection. The Wilcoxon test was used for statistical comparisons of amounts of sucrose consumption before and after CTA training (**P < 0.01, ns: not significant).

In the group that consumed sucrose solution 5–10 min before LiCl injection (5–10 min before group), sucrose consumption was significantly reduced in the final post-training test compared to that in the initial test (n = 21, Wilcoxon test, p = 0.044, Fig. 3). On the other hand, in the other groups, sucrose consumption in the final test did not significantly differ from that in the initial test (Wilcoxon test, 8 h before group: n = 31, p = 0.27; 1 h before group: n = 15, p = 0.92; 5–10 min after group: n = 17, p = 0.33; 1 h after group: n = 19, p = 0.52; 8 h after group: n = 18, p = 0.61). In short, crickets exhibited reduced sucrose consumption when the crickets consumed sucrose 5–10 min before LiCl injection but not when crickets consumed 5–10 min after LiCl injection or when the interval was 1 h or longer. This finding indicates that reduced sucrose consumption of the group that consumed sucrose 5–10 min before LiCl injection is not due to a non-associative, general toxic effect of LiCl to reduce appetite, because all other groups that received injection of LiCl with the same interval between injection and the final test exhibited no reduction of sucrose consumption in the final test. We thus conclude that reduction in consumption of sucrose solution of the group that consumed sucrose 5–10 min before LiCl injection is specific to CS-US pairing, and hence taste aversion conditioning by pairing ingestion of sucrose solution with LiCl injection is successful.

### Retention for 48 h after CTA training with sucrose or fructose

We next investigated whether the memory formed by one-trial CTA training can be maintained for 48 h and we also investigated whether CTA can be achieved with fructose as the CS. Two groups of crickets received an initial consumption test of 0.5 M sucrose and the next day they were given 100 µl of 0.5 M sucrose solution at 8 h or 5–10 min before injection of 5 µl of 1 M LiCl solution. At 48 h after LiCl injection, they received the final consumption test. Another two groups received the same training and testing with 1 M fructose solution.

**Figure 4:**
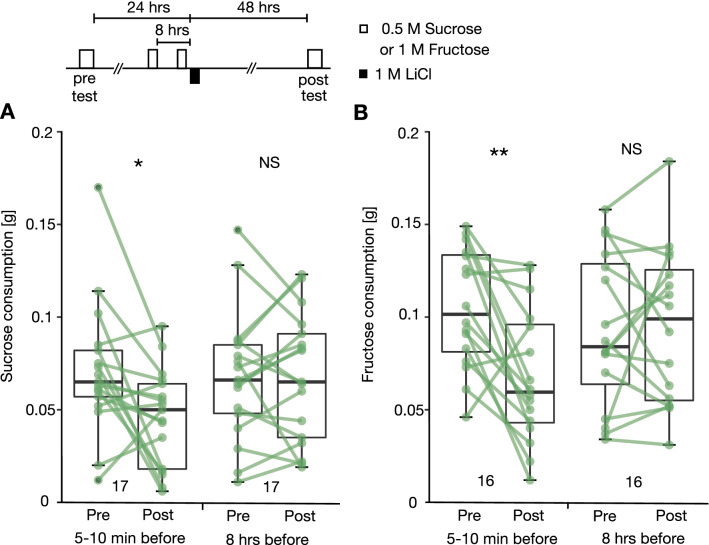
Effects of CTA training with sucrose or fructose solution tested 2 days after LiCl injection. (**A**) Two groups of crickets received a 0.5 M sucrose consumption test, and the next day they were allowed to ingest 100 µm of 0.5 M sucrose solution 5–10 min or 8 h before LiCl injection. Their sucrose consumption was tested again 48 h after LiCl injection. (**B**) Another two groups received CTA training and testing with 1 M fructose solution. The Wilcoxon test was used for statistical comparisons of amounts of sugar consumption 24 h before and 48 h after CTA training (*P < 0.05, **P < 0.01, ns: not significant).

The groups that consumed sucrose or fructose solution 5–10 min before LiCl injection exhibited significant reduction of sucrose or fructose consumption in the final test compared to that in the initial test (Wilcoxon test, sucrose: n = 17, p = 0.023; fructose: n = 16, p = 0.007, Fig. 4A, B). On the other hand, the group that ingested sucrose or fructose solution 8 h before LiCl injection exhibited no significant reduction of sucrose or fructose consumption in the final test compared to that in the initial test (Wilcoxon test, sucrose: n = 17, p = 0.89; fructose: n = 16, p = 0.98, Fig. 4A, B). We thus conclude that the memory formed by one-trail CTA conditioning with sucrose or fructose solution is retained for at least 48 h.

## Discussion

### Conditioned taste aversion in crickets

We have established procedures to achieve CTA in crickets. In training, crickets were allowed to ingest sucrose solution and were then injected with LiCl into the haemolymph 5–10 min later. Crickets that received single-trial CTA training exhibited a significant reduction of sucrose consumption in the test performed 24 or 48 h after LiCl injection compared to that in the initial test performed 24 h before LiCl injection. In contrast, crickets that received a pairing trial in which sucrose was given 5–10 min after LiCl injection or the interval between sucrose consumption and LiCl injection was 1 h or longer exhibited no reduction of sucrose consumption in the final test compared to that in the initial test. The results indicate that reduction of sugar consumption is due to pairing of it with subsequent LiCl injection, not due to a non-specific toxic effect of LiCl to reduce motivation to intake fluid. Single CTA training with fructose solution was also successful, with memory being retained for 2 days. We thus conclude that single pairing of sugar ingestion with LiCl injection is sufficient to achieve CTA and that the memory is retained for at least 2 days in crickets. Our findings in crickets are in accordance with results of previous studies on CTA in locusts (*Schistocerca americana*) showing that a single session of training with spinach or broccoli and then injection of a toxin was sufficient to achieve aversion to spinach or broccoli and that the memory was retained for 2 days^15,16^.

We found that CTA is successful with a 5–10-min interval between sucrose ingestion and LiCl injection in crickets. This interval is much longer than that to achieve an association between CS and US in most Pavlovian conditioning systems in insects. For example, we have observed that conditioning of odor CS with water US in crickets is difficult to achieve when the CS-US interval is 20 s or longer^28^. Association of CS and US with long intervals is a characteristic feature of CTA in animals, indicating that a negative physiological consequence of ingestion of toxin occurs with long latencies after ingestion of toxin-containing food.

CTA was not successful when the interval between sucrose consumption and LiCl injection was 1 h or longer. This differs from reports for rats showing that CTA is achieved with intervals of several hours between food consumption and LiCl injection^6^. Such a difference between insects and mammals can be explained if malaise occurs sooner after ingestion of toxin-containing food in insects due possibly to the smallness and simplicity of the digestive system, and hence avoidance of the taste of food that was ingested long before the occurrence of malaise is less important in insects. To clarify this issue, physiological mechanisms to monitor malaise signals and neural mechanisms to associate it with the taste of previously ingested food need to be investigated in insects to compare them with those in mammals. In this respect, a recent finding that serotonin is likely to mediate malaise signals in honey bees^17,20,21^ is promising as a basis for future studies.

### Comparison to odor aversion learning in locusts

Simões et al.^22^ investigated aversive learning of food odor by pairing food ingestion with toxin injection in locusts (*Schistocerca americana*) and found that learning is achieved with an interval between food uptake and toxin injection of 30 min but not with an interval of 1 h, and their results show some agreement with our findings for taste aversion learning in crickets. Since it has been reported that odor learning occurs only when an odor is presented during food uptake^22^, it can be reasoned that that sensory stimuli during food uptake, most probably taste, serve as critical cues for achieving conditioning of odor with malaise. One of the possible mechanisms is that food taste mediates the association between odor and malaise by the mechanism of second-order conditioning, i.e., conditioning of taste with malaise is coupled with conditioning of odor with taste as suggested in rats^7^. Food-odor aversion by uptake of toxin-containing food has also been reported in some other species of insects (honey bees^17,18,20,21^ and fruit-flies^19,29^). and it would be interesting to investigate if these are also due to second-order conditioning.

### Comparison to the nature of CTA in snails

The nature of CTA in crickets also differs from that in snails (*Lymnaea stagnalis*). In snails, repetitive pairings of sucrose solution and KCl produce suppression of feeding response to sucrose solution and the memory lasts longer than one month^12^. Sugai et al.^11^ reported that one-trial conditioning produces memory that lasts for 5 days in some but not all individuals. Nakai et al.^13^ reported that successful conditioning is achieved with intervals between sucrose and KCl of 10 s to 3 min. How different features of CTA among different animals reflects different feeding habits and different strategies to avoid toxin ingestion remains as an interesting future subject.

### Future perspectives and conclusions

This study was designed to evaluate the effect of CTA training based on within-group statistical comparisons, in which the amount of sucrose consumption after training of individual crickets was compared with that before training. We did not use statistical comparisons between groups, because the amount of sucrose consumption before training was highly variable among individuals and hence statistical comparisons of the amount of sugar consumption after training in different individuals were less effective to obtain statistical conclusions. Improvements of experimental procedures are needed for allowing between-group statistical comparisons and for achieving detailed analysis of data.

One of the reasons for the establishment of a CTA procedure is that we intend to use this procedure for “US devaluation” experiments that will allow us to characterize associative processes that govern execution of a conditioned response (CR) after Pavlovian conditioning. We previously showed that after a standard amount of Pavlovian training to associate an odor CS with water US, crickets exhibited no CR when water US was devalued by providing it until satiation^30,31^. After extended training, on the other hand, the level of CR remained unchanged by devaluation of the US. This finding suggests that execution of a CR is initially governed by the current value of the US, but it becomes more automatic and independent of the current US value^30,32^. Such an increase of behavioral automaticity by extended Pavlovian training has not been reported in mammals^33,34^ or in any other animals as we discussed in our previous studies^30,32^, and hence we think that evaluation of the reproducibility of the results obtained by the use of CTA for devaluation of the US is needed. Indeed, a CTA procedure has been successfully used for devaluation of sucrose US in honey bees^17^, in which bees that had subjected to pairing of an odor CS with sucrose US and then subjected to pairing of sucrose with toxin (quinine) exhibited partially reduced response to the odor CS, indicating that the execution of the CR depends partially on the current value of the US.

In conclusion, we showed that CTA in crickets has a feature similar to that in mammals in that one-trial CTA training is sufficient for producing long-term aversive memory that is retained for at least two days, but it also has a different feature in that CTA in crickets is not achieved with a long interval between sugar intake and toxin injection of 1 h or longer. Insects are useful animals for analyzing the mechanisms of CTA due to the simplicity of their central nervous system, and recent findings in honey bees indicating that malaise signals produced by toxin are likely to be mediated by serotonin^17,20,21,35^ are promising as the first step for such studies. Since various experimental manipulations such as pharmacological analysis, RNAi and genome editing by CRISPR/Cas9 are feasible in crickets^23,24,27^, further studies in crickets are promising to extend our understanding of the physiological and neural mechanisms of CTA.

## Materials and methods

### Insects

A wild-type strain of two-spotted crickets (*Gryllus bimaculatus*) has been inbred for several decades in our laboratory (Hokudai WT strain). The crickets were reared in 12-h light/dark cycles at 29 °C ± 2 °C and were fed a diet of insect pellets and water ad libitum. Three days after the imaginal molt, adult male crickets were individually isolated in 100 ml glass beakers. They were given insect pellets ad libitum but were deprived of water to enhance motivation to uptake liquid. Crickets weighting 0.69 ± 0.09 (mean ± SD, n = 165) g were used.

### Survival analysis

To determine the appropriate concentration of LiCl solution for use in a CTA experiment, three groups of crickets were allowed to consume 0.5 M sucrose from a feeder for 2.5 min and they were injected 5 min later with 5 μl of saline or saline containing 1 M or 2 M LiCl solution. The survival probabilities of the three groups were recorded at 4, 8, 12, 24, 36 and 48 h after injection.

### Procedures for taste aversion conditioning

In a typical conditioning experiment, 100 µl of 0.5 M sucrose or fructose solution was attached to the wall of the beaker in which a cricket was placed and the cricket was allowed to consume it. Crickets typically consumed it within 5 min. Crickets that did not complete consumption of 100 µl of sugar solution within 15 min were not used for experiments, considering the possibility that they have a poor physiological condition. Those crickets accounted for 24% (39/260) of the crickets used in this experiment. At 5–10 min after sugar consumption, they were injected with 5 μl of cricket saline^36^ or saline containing 1 M LiCl by inserting a microsyringe into the ventral side of the thorax. The dose per body weight of crickets was 0.31 ± 0.03 (mean ± SD, n = 165) mg/g. In experiments in which LiCl was injected at various times before and after providing sugar solution, LiCl injection was set at the onset of the dark phase of 12 h:12 h light–dark cycles.

### Consumption tests

The amount of consumption of sugar solution was measured by scaling the weights of the feeder before and after the test (Fig. 1). This was calibrated by the rate of evaporation, although its contribution was negligible. The feeder, composed of a plastic petri dish of 30 mm in diameter and a bottle cap (Fig. 1A), was placed in the center of an acrylic cubic box measuring 15 cm × 15 cm × 15 cm (Fig. 1B). The test started when a cricket was placed in the box. The cricket was allowed to freely visit the feeder for a period of 2.5 min.

### Statistical Analysis

The survival probabilities of the three groups of crickets that had been injected with different concentrations of LiCl solution were assessed by using Kaplan–Meier survival analysis^37^, and the log-rank test was use for comparing survival probabilities of different groups using GraphPad Prism 9. Because of the increased risk of a Type I error when performing multiple statistical tests, we applied Bonferroni’s correction. The Wilcoxon signed-rank test was used for comparing the amount of consumption of sugar solution in a test performed after training with that in a test performed before training for each group. Statistical analyses were performed using R 3.6.1 (Package: ggplot2 version 3.2.0, ggpubr version 0.2.1). P < 0.05 was considered as a significant difference.

## Data Availability

The datasets generated during the current study are available from the corresponding author on reasonable request.
